# Supplementary material for: Concomitant Nrf2- and ATF4-Activation by Carnosic Acid Cooperatively Induces Expression of Cytoprotective Genes
Source: Int J Mol Sci. 2019 Apr 5;20(7):1706. doi: 10.3390/ijms20071706 (PMC6480217; doi:10.3390/ijms20071706)
Supplement: Supplementary file 1 [file ijms-20-01706-s001.zip › Supplementary Table S2.docx]

**Supplementary Table S2.
Primers used for RT-qPCR analysis**

| gene name | forward primer | reverse primer |
| --- | --- | --- |
| *hNGF* | 5’-CCAAGGGAGCAGCTTTCTATCCTGG-3’ | 5’-GGCAGTGTCAAGGGAATGCTGAAGT-3’ |
| *hCyclophilin A* | 5’-ATGCTGGACCCAACACAAAT-3’ | 5’-TCTTTCACTTTGCCAAACACC-3’ |
| *hHO-1/HMOX1* | 5’-CCAGCAACAAAGTGCAAGATTC-3’ | 5’-TCACATGGCATAAAGCCCTACAG-3’ |
| *hATF4* | 5’-TCTCCAGCGACAAGGCTAA-3’ | 5’-CAATCTGTCCCGGAGAAGG-3’ |
| *hASNS* | 5’-CCAATTCGAGTGAAGAAATATCC-3’ | 5’-TTCAAATTCAAAATGCTGTTGC-3’ |
| *hTrib3* | 5’-CCGTCTTGGGCCCTATGT-3’ | 5’-GTACCAGCCAGGACCTCAGT-3’ |
| *hNrf2* | 5’-GCAACAGGACATTGAGCAAG-3’ | 5’-TGGACTTGGAACCATGGTAGT-3’ |
| *hTXNRD1* | 5’-ACACAAAGCTTCAGCATGTCA-3’ | 5’-CAATTCCGAGAGCGTTCC-3’ |
| *hAKR1B10* | 5’-AGATGATAAAGGTAATGCCATCG-3’ | 5’-TGGAAGTGGCTGAAATTGG-3’ |
| *hxCT/SLC7A11* | 5’-CCATGAACGGTGGTGTGTT-3’ | 5’-GACCCTCTCGAGACGCAAC-3’ |
| *hAKR1C1&2* | 5’-TGGGATTTGGCACCTATGCG-3’ | 5’-CCTGGCTTGTTGAGGATCAT-3’ |
| *hNQO1* | 5’-GTCATTCTCTGGCCAATTCAGAGT-3’ | 5’-TTCCAGGATTTGAATTCGGG-3’ |
| *hGCLM* | 5’-TGGGCACAGGTAAAACCAA-3’ | 5’-CAGTCAAATCTGGTGGCATC-3’ |
| *hGCLC* | 5’-TTGACGATAGATAAAGAGATCTACGAA-3’ | 5’-TCTCTAATAAAGAGATGAGCAACATGC-3’ |
| *hp62* | 5’-AGCTGCCTTGTACCCACATC-3’ | 5’-CAGAGAAGCCCATGGACAG-3’ |
| *hSESN2* | 5’-ACTGCGTCTTTGGCATCAG-3’ | 5’-GTAGCAGGCCACTGTCTTGA-3’ |
