# Supplementary material for: Concomitant Nrf2- and ATF4-Activation by Carnosic Acid Cooperatively Induces Expression of Cytoprotective Genes
Source: Int J Mol Sci. 2019 Apr 5;20(7):1706. doi: 10.3390/ijms20071706 (PMC6480217; doi:10.3390/ijms20071706)
Supplement: Supplementary file 1 [file ijms-20-01706-s001.zip › Legends for Supplementary Figures rev.3.docx]

Supplementary Figure legends

**Supplemental Figure S1.**

**CA induction of ATF4 in other cancer cell lines and ATF4 dependency of AARE genes in U373MG cells.**

(A) HeLa, (B) Caco-2 and (C) HepG2 cells were treated with increasing amount of CA as indicated in figure for 6 h, then an aliquot of nuclear extract was subjected to immunoblot analysis as Figure 2C. (B to I) The ATF4-dependent gene inductions of (D) *Trib3* and (E) *CHOP*, (F) *CBS*, (G) *CTH* (H) *SESN2* and (I) *CHAC1* in U373MG cells were demonstrated by RT-qPCR as described in Figure 3B. The fold gene expressions were described as mean ± SEM. Open and closed bars indicate DMSO- and 50 μM of CA-treated samples, respectively. The asterisks indicate significant differences between DMSO vs. CA (*, *p* < 0.05, **, *p* < 0.01), and the daggers indicate significant decreases from control siRNA with CA vs Nrf2 and/or ATF4 siRNA with CA (†, *p* < 0.05) by One-way ANOVA with Tukey-Kramer *post hoc* test (*n* = 3).

**Supplemental Figure S2.**

**CA or tBHQ inductions of Nrf2/ARE pathway genes.**

CA- or tBHQ-inducible gene expressions of (A) *HO-1* and (B) *TXNRD1*, (C) *xCT*, (D) *GCLC*, and (E) *GCLM* and (F) *NQO1* in U373MG cells were analyzed as described in Figure 1A. Each value was normalized with cyclophilin A (CypA) expression and presented as the mean ± SEM from three independent experiments. The significant differences from the vehicle control (DMSO) are indicated by asterisks (*, *p* < 0.05, **, *p* < 0.01), the daggers indicate a significant difference from the 50 μM of CA and 50 μM of tBHQ (†, *p* < 0.05, ††, *p* < 0.01, NS: not significant) by one-way ANOVA with Tukey-Kramer *post hoc* test (*n* = 3).

**Supplemental Figure S3.**

**Different Nrf2- and ATF4-dependency of Nrf2/ARE pathway genes.**

RT-qPCR analysis for (A) *xCT*, (B) *p62*, (C) *GCLC*, (D) *GCLM* and (E) *NQO1* in ATF4 and/or Nrf2 knockdown cells were performed as described in Figure 3B. The fold gene expressions were described as mean ± SEM. Open and closed bars indicate DMSO and CA treated samples, respectively. The asterisks indicate significant differences between DMSO vs. CA (*, *p* < 0.05, **, *p* < 0.01), and the daggers indicate significant decreases from control siRNA with CA vs Nrf2 and/or ATF4 siRNA with CA (†, *p* < 0.05, ††, *p* < 0.01), and the section signs indicate significant differences from Nrf2/ATF4 double knocked down cells with CA. (§§, *p* < 0.01) by One-way ANOVA with Tukey-Kramer *post hoc* test (*n* = 3).

**Supplemental Figure S4.**

**Cooperative induction of *NGF* and *HO-1* by tBHQ and Tm in NHAs.**

(A) *NGF* and (B) *HO-1* gene expressions in response to 50 μM of tBHQ and 1 μg/mL Tm in normal human astrocytes (NHAs). The values are presented as the mean ± SE. The asterisks indicate significant differences from the DMSO-treated sample (** *p* < 0.01 by one-way ANOVA with Tukey-Kramer post hoc test). The daggers indicate significant decreases from tBHQ/Tm co-treated sample († *p* < 0.05; †† *p* < 0.01 by one-way ANOVA with Tukey-Kramer post hoc test (*n* = 3)).

**Supplemental Figure S5.**

**CA induction of ATF4 in eIF2α mutant MEFs.**

(A) Mouse embryonic fibroblasts (MEF) harboring eIF2α (Ser51Ala) mutation (MEF(A/A)) or corresponding WT MEF (MEF(S/S)) were treated with 10 to 50 μM of CA for 4 h. After subcellular fractionation, aliquots of nuclear or cytosolic fractions were subjected to immunoblot analysis to evaluate ATF4 protein induction. (B and C) MEF(S/S) and MEF(A/A) were treated with 50 μM of CA for 4 h, then subjected to RT-qPCR analysis to evaluate *Atf4* (B) and *Asns* (C) mRNA expressions.

**Supplemental Figure S6**

**Knockdown efficiencies of eIF2α kinases in U373MG cells.**

U373MG cells were transfected with control (Ctrl) or HRI (A), PKR(B), PERK (C) and GCN2 (D) siRNAs as described in Materials and Methods. After 24 h transfection, the cells were treated with DMSO, 50 μM of CA or 2 μg/mL Tm for 6 h. An aliquot of cytosolic extracts was then subjected to SDS-PAGE and immunoblot analysis for HRI (A), PKR (B), PERK (C) and GCN2 (D). (E) RT-qPCR analysis of HRI siRNA transfected U373MG cells treated with DMSO or 50 μM of CA for 24h. Open and closed bars indicate DMSO and CA treated samples, respectively. The relative gene expressions were described as mean ± SEM. The asterisks indicate significant differences between DMSO and CA treated samples. (* *p* < 0.05; ** *p* < 0.01 by Student’s t-test (n = 3)). The daggers indicate significant decreases between control siRNA/CA and HRI siRNA/CA. (†† *p* < 0.01 by Student’s t-test (*n* = 3)). NS: not significant.

**Supplemental Figure S7**

**Effect of CA on Xbp1 splicing and *HSPA5*/*BiP* induction.**

A-B U373MG cells were treated with DMSO, 50 μM of CA or 2 μg/mL tunicamycin (Tm) for 6 h, and then total RNA was prepared for cDNA synthesis. Xbp1 transcript was amplified by using specific primer pair (5’-TTA CGA GAG AAA ACT CAT GGC-3’ and 5’-GGG TCC AAG TTG TCC AGA ATG C-3’), which discriminate Xbp1 splicing. (B) U373MG cells were treated with DMSO, 50 μM of CA or 2 μg/mL Tm for 24 h. HSPA5/Grp78/BiP gene expression was evaluated by RT-qPCR. The asterisks indicate significant differences between vehicle control (0). (* *p* < 0.05 by One-way ANOVA with Tukey-Kramer *post hoc* test (n = 3)). NS: not significant.

**Supplementary Figure S8.**

**CA activates ISR through HRI.**

(A) The effect of HRI knockdown on CA induction of eIF2α phosphorylation. U373MG cells were transfected with control, HRI#1, or HRI#2 siRNA and incubated for 24 h. The transfected cells were treated with 50 μM of CA for 2 h, and cytosolic extracts were then subjected to immunoblot analysis. (B) Nascent protein synthesis in HRI knocked down cells. HeLa cells were transfected with control or HRI#1 or HRI#2 siRNA, and then subjected to nascent protein labeling as Figure 5B. (C) Representative immunoblots of phosphorylated eIF2α after 1 h treatment with DMSO or 50 µM CA in the six types of MEFs (WT, 4KO, and 4KO with respective FLAG-tagged eIF2α kinases). (D) The effect of hemin on CA-inducible ATF4 and Nrf2 inductions. U373MG cells were pretreated with or without 5 or 10 μM hemin for 30 min, and the cells were then administered DMSO or 50 μM of CA for 6 h. Nuclear extract aliquots were subjected to SDS-PAGE and immunoblot analysis. (E) Biotinylated CA (Bio-CA) pull-down assay. Bio-CA bound proteins were separated and blotted with anti-Keap1 or anti-HRI antibodies. Input corresponds to 10% of the total cell lysates for pull-down. (F) HRI degradation by CA. U373MG cells were administered 50 μM of CA for 1 to 24 h as indicated. Equal amounts of cytosolic extracts were subjected to immunoblot analysis. Closed and open triangles indicate native or slower-migrating HRI bands, respectively.

**Supplemental Figure S9**

**Nrf2 or ATF4 inducibility of CA-related chemicals.**

(A) Conversion of catechol-CA to quinone-CA by oxidation. The asterisk indicates electrophilic carbon atom, which reacts with thiol group (See ref [9]). (B) ATF4 and Nrf2 protein inductions by CA and CA related chemicals (carnosol (CS), rosmarinic acid (RA), piciferic acid (PA)) in U373MG cells. U373MG cells were treated with 50 μM of CA or related chemicals for 6 h. An aliquot of nuclear extract was subjected to SDS-PAGE and then blotted with anti-ATF4, Nrf2 and lamin B antibodies. (C) Chemical structures of CA related chemicals.
