# Supplementary material for: Concomitant Nrf2- and ATF4-Activation by Carnosic Acid Cooperatively Induces Expression of Cytoprotective Genes
Source: Int J Mol Sci. 2019 Apr 5;20(7):1706. doi: 10.3390/ijms20071706 (PMC6480217; doi:10.3390/ijms20071706)
Supplement: Supplementary file 1 [file ijms-20-01706-s001.zip › Supplementary Table S1.docx]

**Supplemental Table S1.
The list of genes up-regulated more than 2.5-fold in 50 μM CA-treated U373MG cells.**

| **Gene Symbol** | **RefSeq_ID** | **Description** | **Dependency*** | | **Fold increase** |
| --- | --- | --- | --- | --- | --- |
|  |  |  | **Nrf2** | **ATF4** |  |
| *AKR1B10* | NM_020299 | Aldo-keto reductase family 1 member B10 | ✓ |  | 18.8 |
| *HMOX1* | NM_002133 | Heme oxygenase 1 | ✓ | ✓ | 12.0 |
| *AKR1C2* | NM_205845 | Aldo-keto reductase family 1 member C2 | ✓ |  | 7.5 |
| *AKR1C1* | NM_001353 | Aldo-keto reductase family 1 member C1 | ✓ |  | 7.3 |
| *HIST3H3* | NM_003493 | Histone H3.1t |  |  | 7.1 |
| *SCRT1* | NM_031309 | Transcriptional repressor scratch 1 |  |  | 5.8 |
| *TXNRD1* | NM_003330 | Thioredoxin reductase 1 | ✓ | ✓ | 4.6 |
| *TRIM16* | XM_085722 | Tripartite motif-containing protein 16 |  |  | 4.5 |
| *CYB5R4* | NM_016230 | Cytochrome b5 reductase 4 |  |  | 4.3 |
| *PSAT1* | XM_371677 | Phosphoserine aminotransferase |  | ✓ | 4.2 |
| *ASNS* | NM_183356 | Asparagine synthetase |  | ✓ | 4.1 |
| *SRXN1* | NM_080725 | Sulfiredoxin-1 | ✓ |  | 4.0 |
| *JDP2* | NM_130469 | Jun dimerization protein |  | ✓ | 3.9 |
| *AP1S2* | NM_003916 | AP-1 complex subunit sigma-2 |  |  | 3.9 |
| *OSGIN1* | NM_182981 | Oxidative stress induced growth inhibitor 1 | ✓ |  | 3.8 |
| *ZFAND3* | NM_021943 | AN1-type zinc finger protein 3 |  |  | 3.6 |
| *AKR1C3* | NM_003739 | Aldo-keto reductase family 1 member C3 | ✓ |  | 3.5 |
| *SLC7A11* | NM_014331 | Cystine/glutamate transporter | ✓ | ✓ | 3.5 |
| *ING4* | NM_016162 | Inhibitor of growth protein 4 |  |  | 3.4 |
| *GCLC* | NM_001498 | Glutamate--cysteine ligase catalytic subunit | ✓ | ✓ | 3.4 |
| *CBS* | NM_000071 | Cystathionine beta-synthase |  |  | 3.3 |
| *CXCR7* | NM_020311 | C-X-C chemokine receptor type 7 |  |  | 3.2 |
| *CRYGS* | NM_017541 | Beta crystallin S |  |  | 2.9 |
| *NUPR1* | NM_012385 | Nuclear protein 1 |  | ✓ | 2.9 |
| *MTR* | NM_000254 | Methionine synthase |  |  | 2.9 |
| *AFP* | NM_001134 | Alpha-fetoprotein precursor |  |  | 2.9 |
| *MTHFD2* | NM_006636 | Bifunctional methylenetetrahydrofolate  dehydrogenase/cyclohydrolase, mitochondrial precursor |  | ✓ | 2.8 |
| *GRRP1* | NM_024869 | Glycine/arginine rich protein 1 |  |  | 2.8 |
| *SLC1A5* | NM_005628 | Neutral amino acid transporter B(0) |  | ✓ | 2.8 |
| *KIAA2026* | NM_001017969 | KIAA2026 |  |  | 2.8 |
| *MRPS2* | NM_016034 | Mitochondrial 28S ribosomal protein S2 |  |  | 2.8 |
| *LARS* | NM_020117 | Leucyl-tRNA synthetase, cytoplasmic |  | ✓ | 2.8 |
| *ENTPD2* | NM_203468 | Ectonucleoside triphosphate diphosphohydrolase 2 |  |  | 2.7 |
| *STEAP1* | NM_012449 | Six transmembrane epithelial antigen of prostate 1 |  |  | 2.7 |
| *TARS* | NM_152295 | Threonyl-tRNA synthetase, cytoplasmic |  | ✓ | 2.6 |
| *MARS* | NM_004990 | Methionyl-tRNA synthetase, cytoplasmic |  | ✓ | 2.6 |
| *UCHL3* | NM_006002 | Ubiquitin carboxyl-terminal hydrolase isozyme L3 |  |  | 2.6 |
| *CTH* | NM_001902 | Cystathionine gamma-lyase |  | ✓ | 2.6 |
| *ATF4* | NM_182810 | Cyclic AMP-dependent transcription factor ATF-4 |  | ✓ | 2.6 |
| *PCK2* | NM_004563 | Phosphoenolpyruvate carboxykinase [GTP], mitochondrial precursor |  | ✓ | 2.6 |
| *SUGT1* | NM_006704 | Suppressor of G2 allele of SKP1 homolog |  |  | 2.6 |
| *SLC3A2* | NM_002394 | 4F2 cell-surface antigen heavy chain (4F2hc) | ✓ | ✓ | 2.6 |
| *PDPN* | NM_198389 | Podoplanin precursor |  |  | 2.6 |
| *PGD* | NM_002631 | 6-phosphogluconate dehydrogenase, decarboxylating | ✓ |  | 2.5 |
| *PLAT* | NM_000931 | Tissue-type plasminogen activator precursor |  | ✓ | 2.5 |
| *MDM4* | NM_002393 | Mdm4 protein |  |  | 2.5 |
| *TPD52L1* | NM_003287 | Tumor protein D53 |  |  | 2.5 |
| *TRIB3* | NM_021158 | Tribbles homolog 3 | ✓ | ✓ | 2.5 |

*Nrf2 or ATF4 dependency is based on references ([1], [2], [12], [17])
