# Supplementary figures and images for: Concomitant Nrf2- and ATF4-Activation by Carnosic Acid Cooperatively Induces Expression of Cytoprotective Genes
Source: Int J Mol Sci. 2019 Apr 5;20(7):1706. doi: 10.3390/ijms20071706 (PMC6480217; doi:10.3390/ijms20071706)

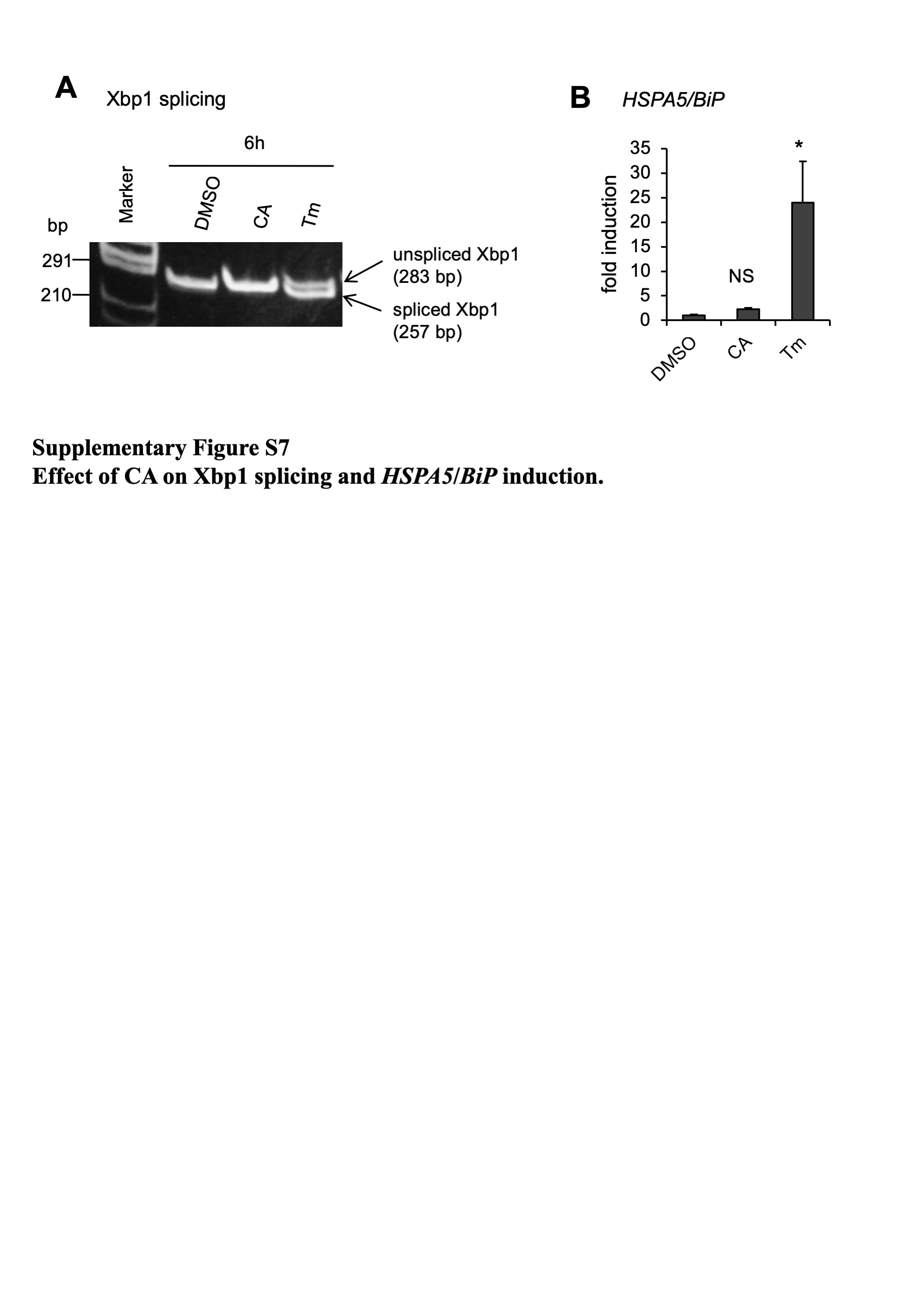

Supplement: Supplementary file 1 [file ijms-20-01706-s001.zip › Supplementary Figure S7.jpeg]

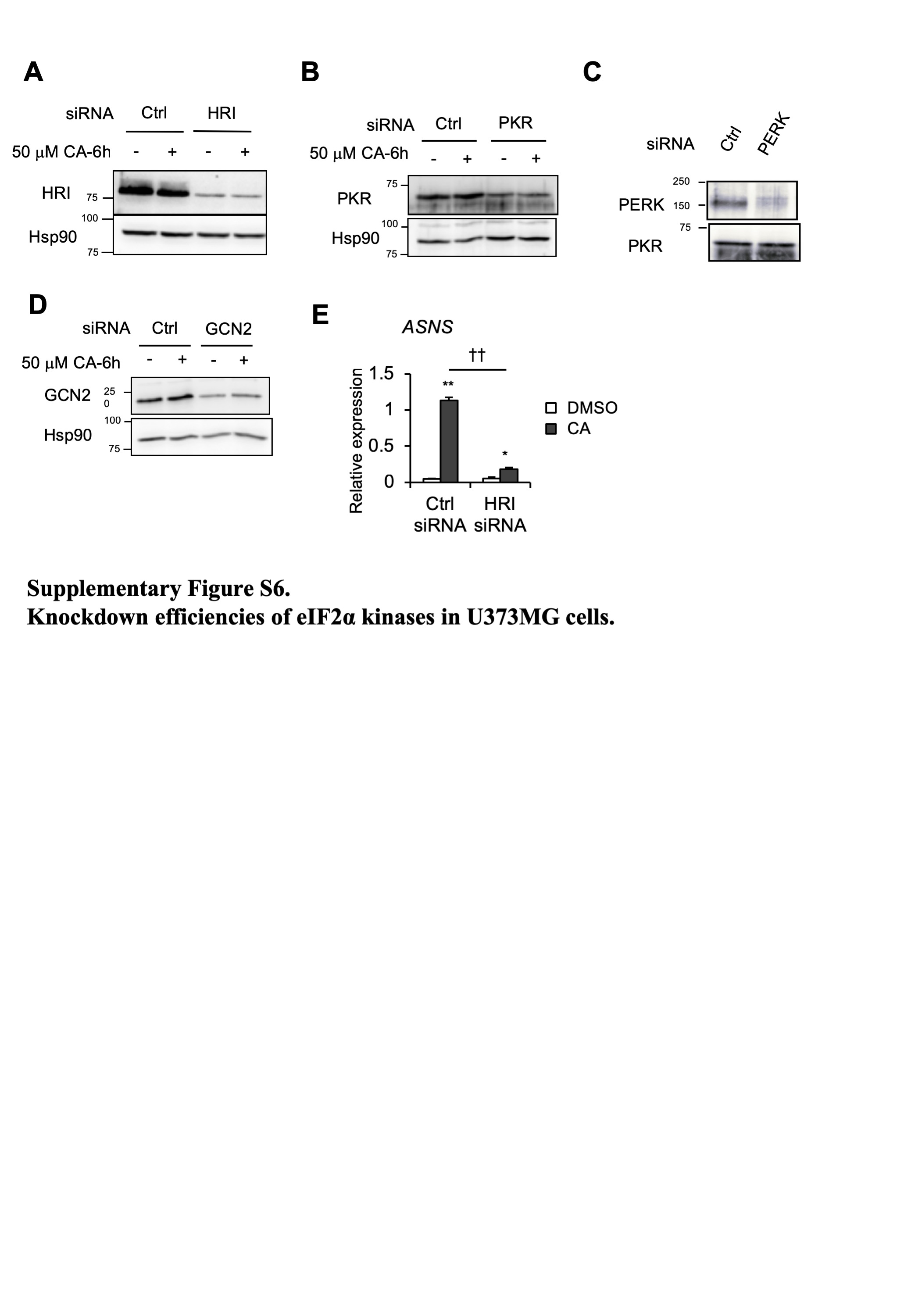

Supplement: Supplementary file 1 [file ijms-20-01706-s001.zip › Supplementary Figure S6.jpeg]

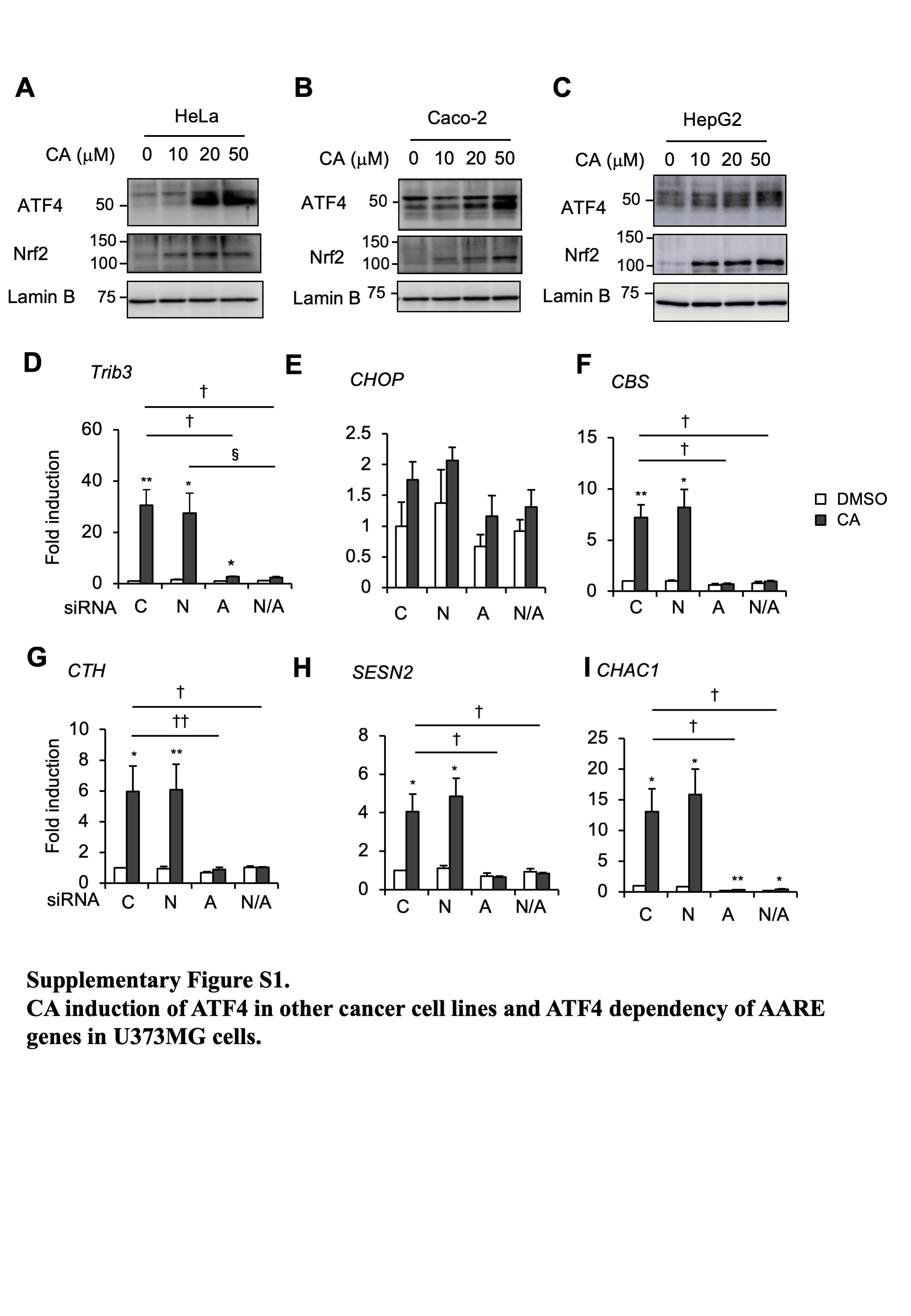

Supplement: Supplementary file 1 [file ijms-20-01706-s001.zip › Supplementary Figure S1.jpeg]

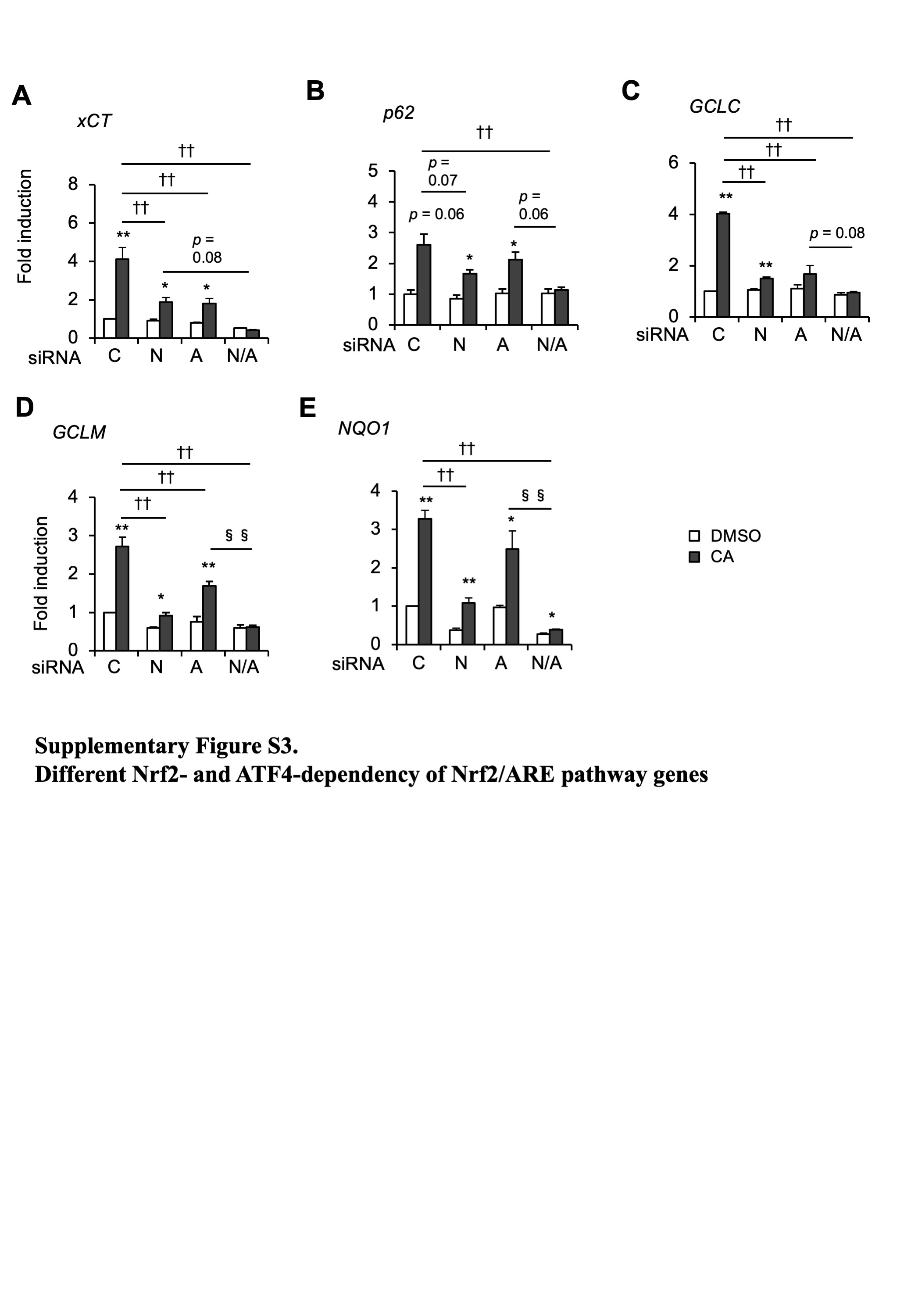

Supplement: Supplementary file 1 [file ijms-20-01706-s001.zip › Supplementary Figure S3.jpeg]

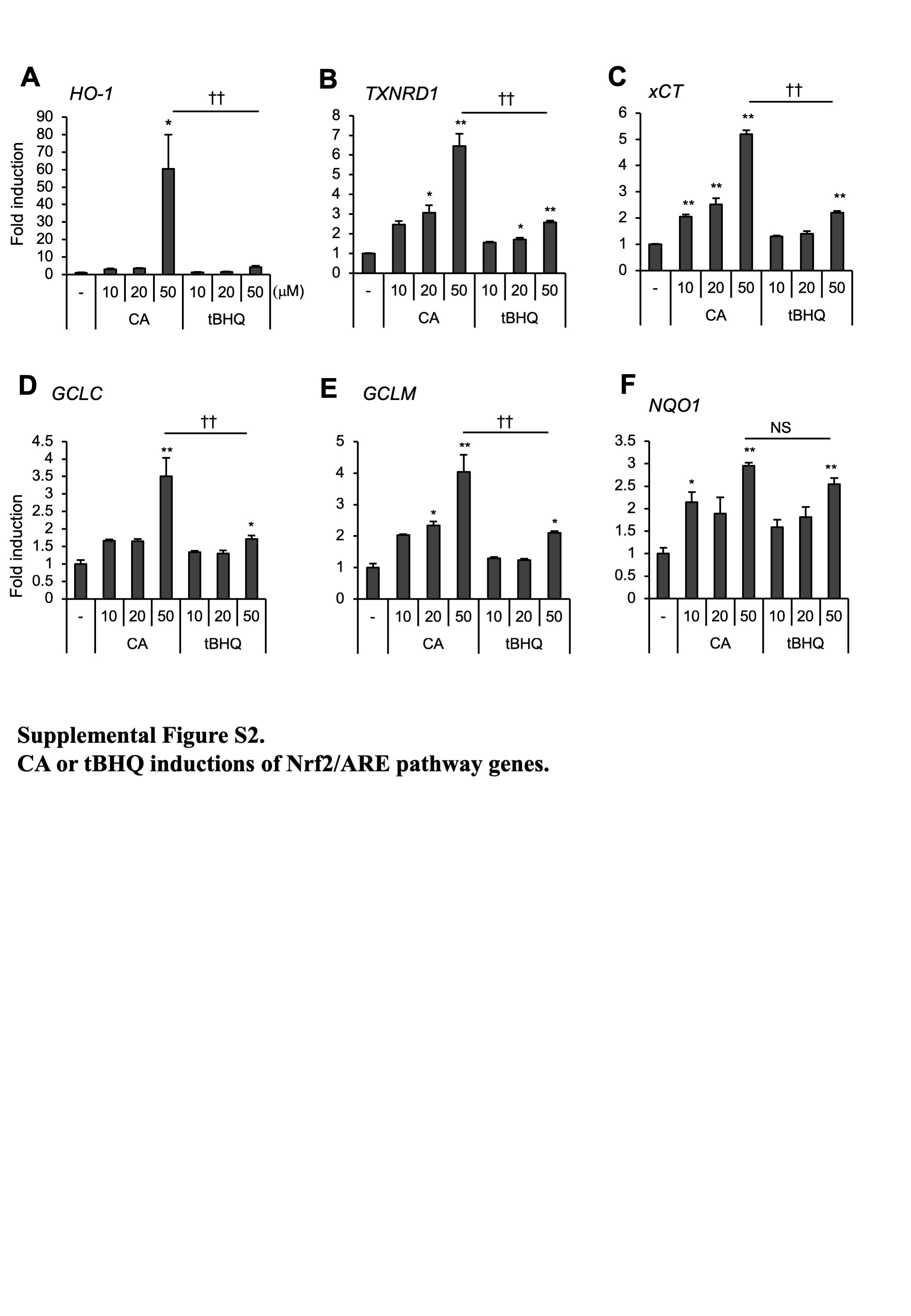

Supplement: Supplementary file 1 [file ijms-20-01706-s001.zip › Supplementary Figure S2.jpeg]

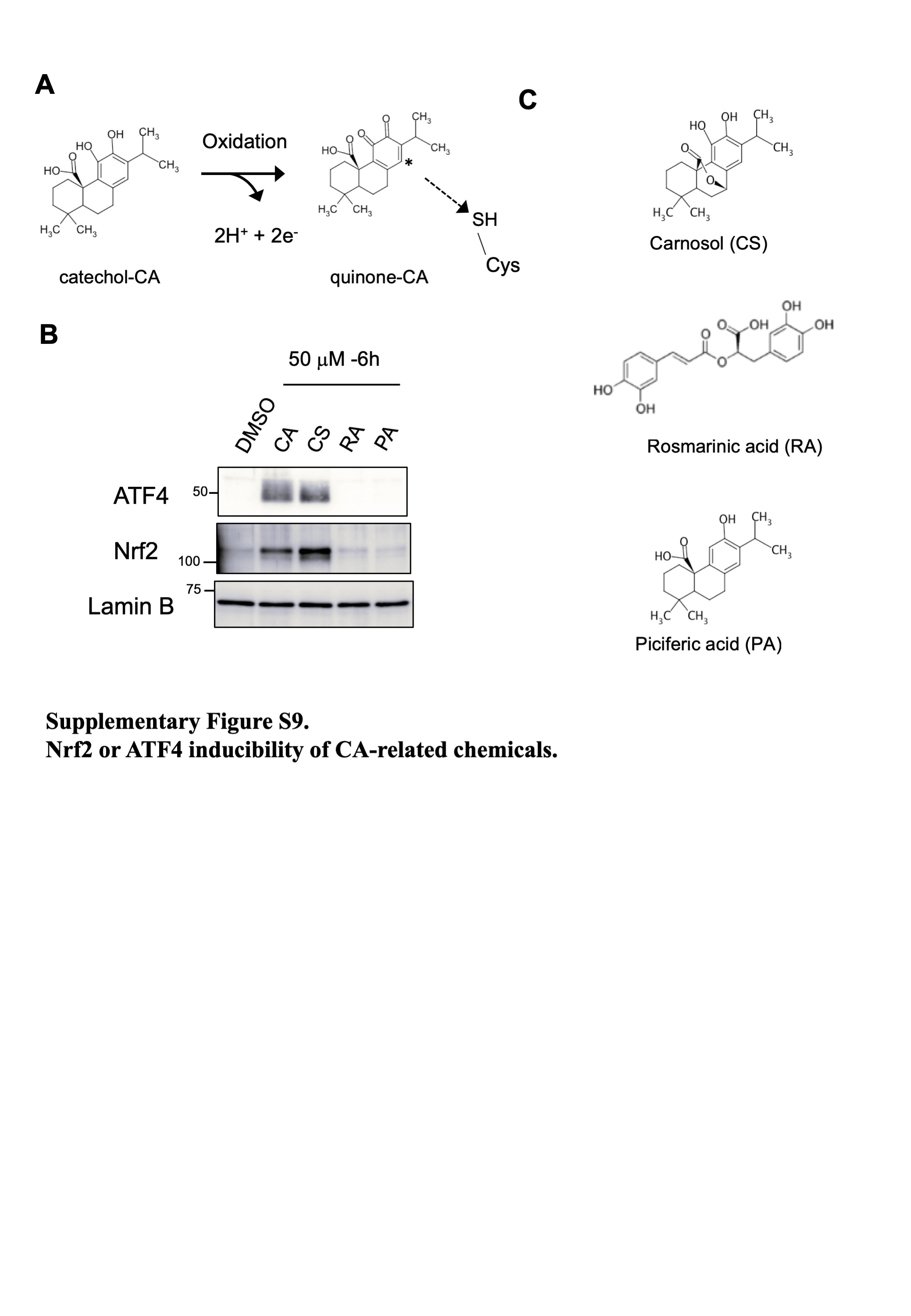

Supplement: Supplementary file 1 [file ijms-20-01706-s001.zip › Supplementary Figure S9.jpeg]

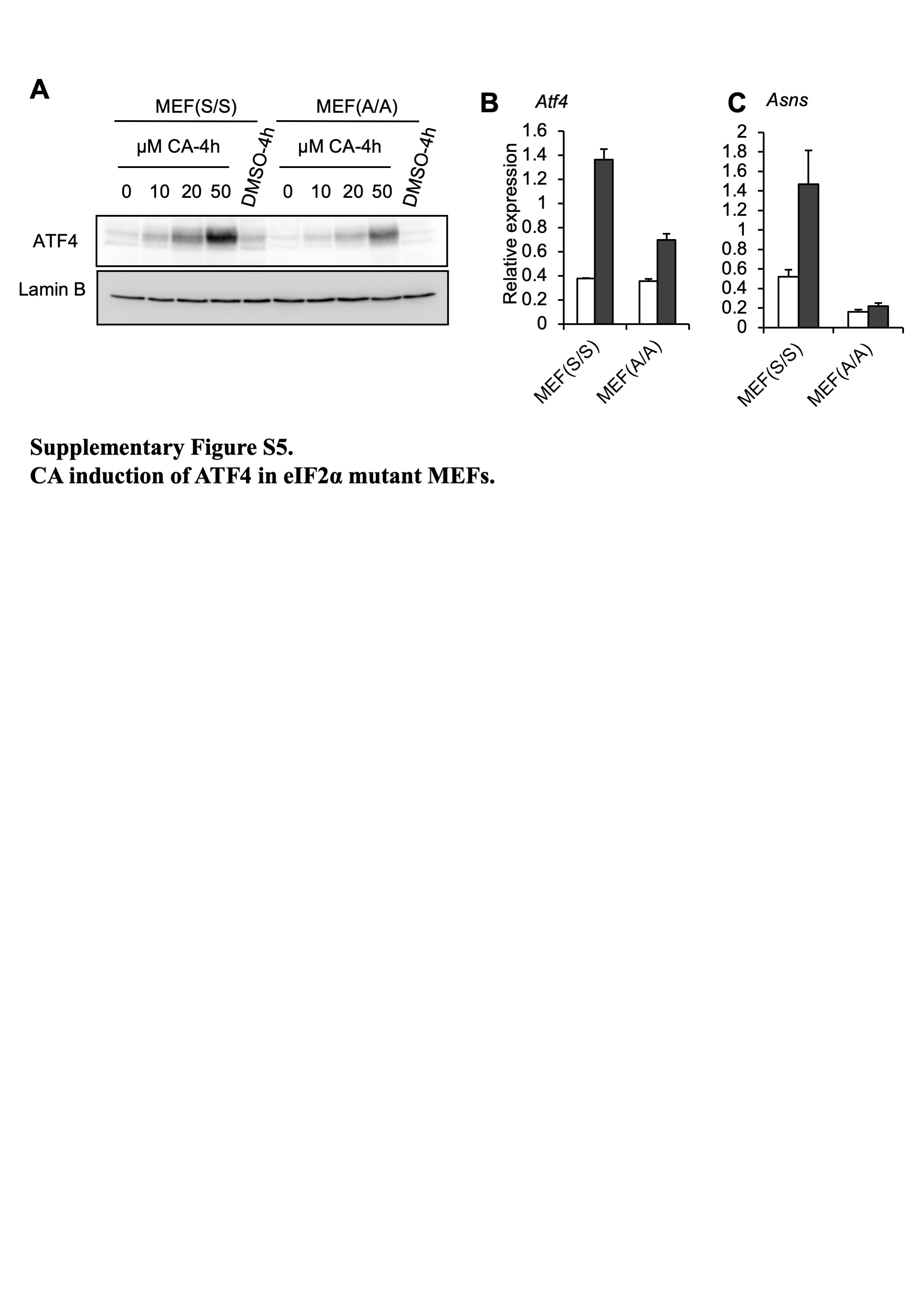

Supplement: Supplementary file 1 [file ijms-20-01706-s001.zip › Supplementary Figure S5.jpeg]

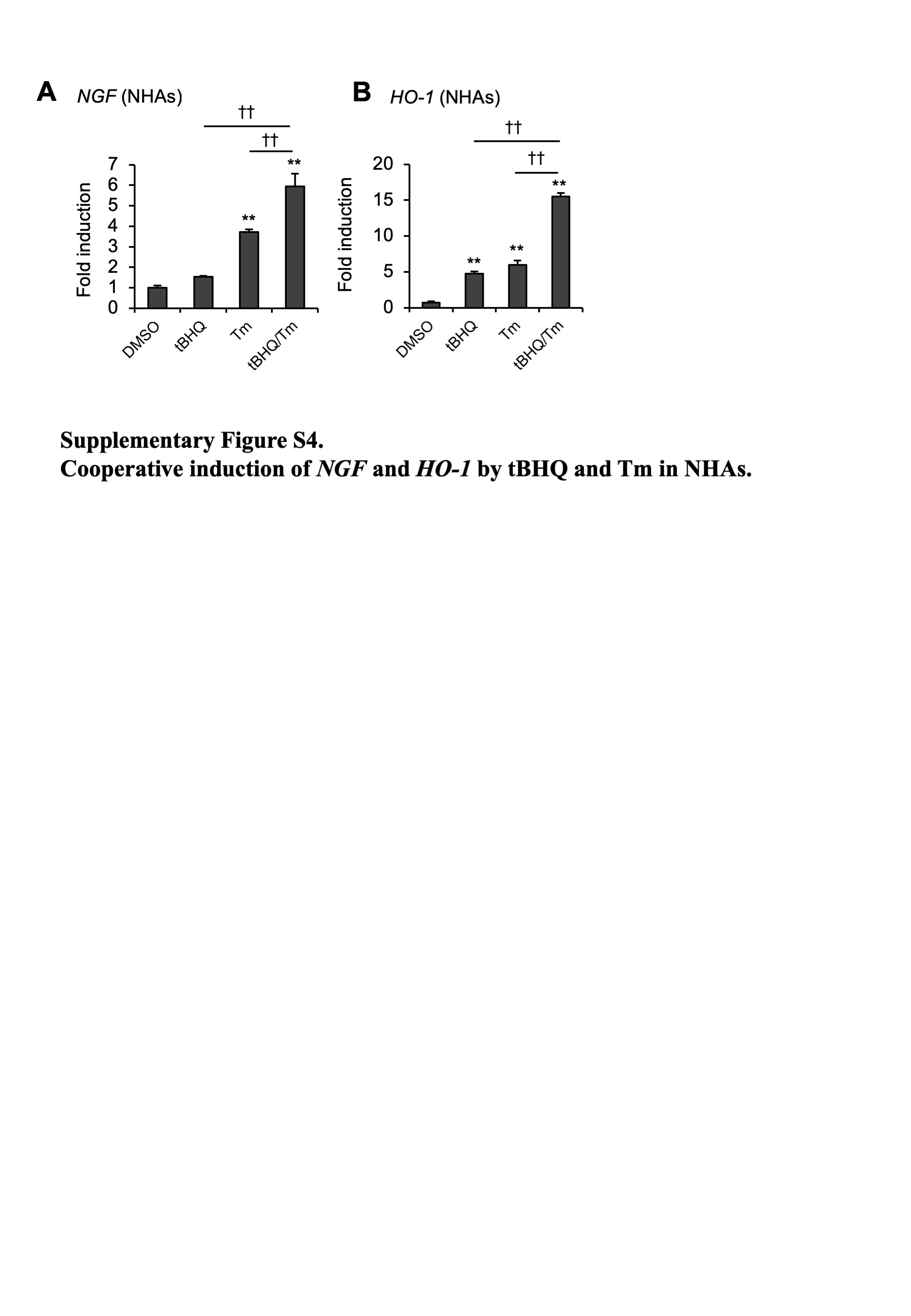

Supplement: Supplementary file 1 [file ijms-20-01706-s001.zip › Supplementary Figure S4.jpeg]

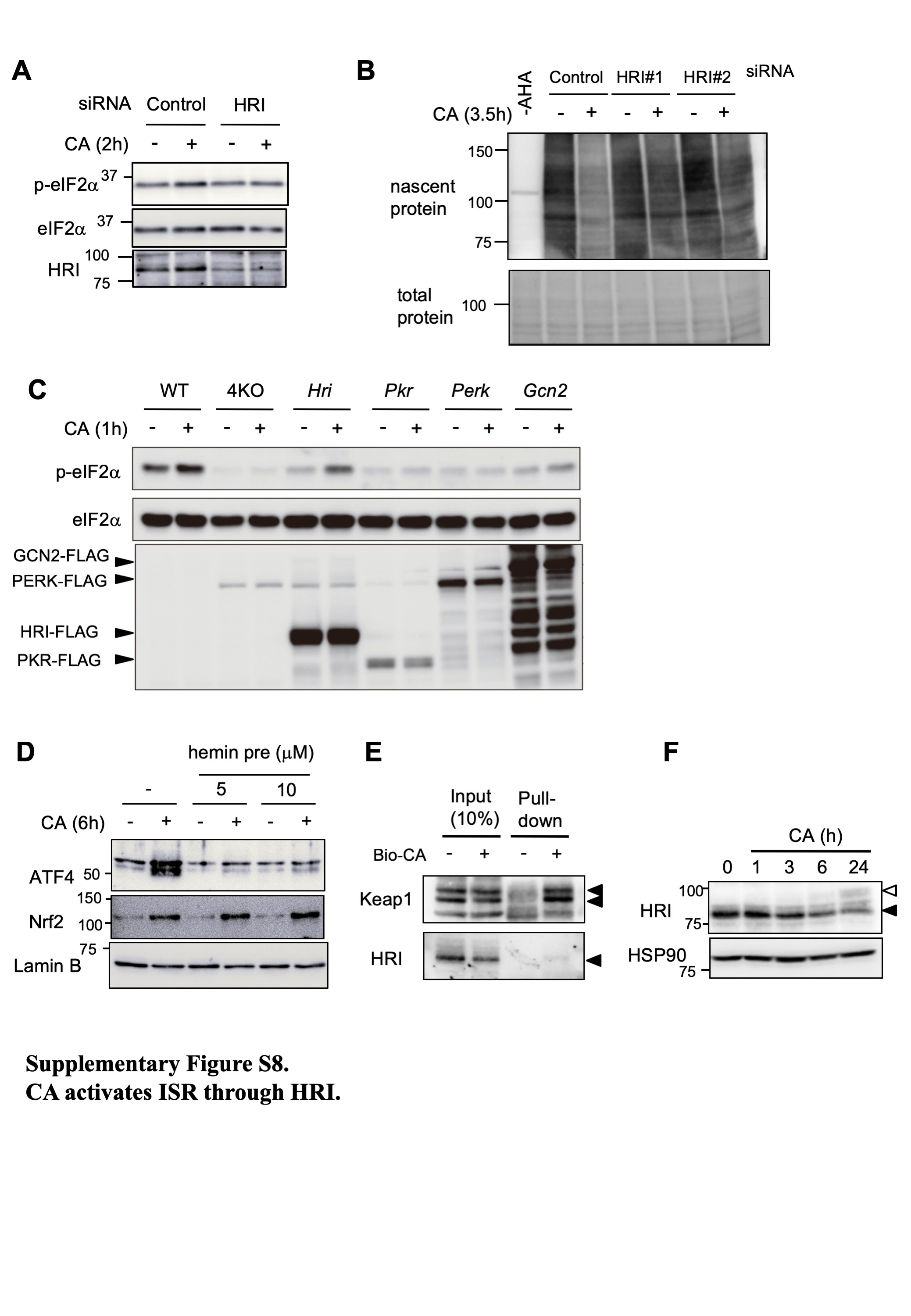

Supplement: Supplementary file 1 [file ijms-20-01706-s001.zip › Supplementary Figure S8.jpeg]
